# Modeling the effects of strigolactone levels on maize root system architecture

**DOI:** 10.3389/fpls.2023.1329556

**Published:** 2024-01-11

**Authors:** Abel Lucido, Fabian Andrade, Oriol Basallo, Abderrahmane Eleiwa, Alberto Marin-Sanguino, Ester Vilaprinyo, Albert Sorribas, Rui Alves

**Affiliations:** ^1^ Systems Biology Group, Department Ciències Mèdiques Bàsiques, Faculty of Medicine, Universitat de Lleida, Lleida, Spain; ^2^ Institut de Recerca Biomèdica de Lleida (IRBLleida), Lleida, Spain

**Keywords:** maize, strigolactones, rsa, root system architecture, mathematical model, multiscale modeling

## Abstract

Maize is the most in-demand staple crop globally. Its production relies strongly on the use of fertilizers for the supply of nitrogen, phosphorus, and potassium, which the plant absorbs through its roots, together with water. The architecture of maize roots is determinant in modulating how the plant interacts with the microbiome and extracts nutrients and water from the soil. As such, attempts to use synthetic biology and modulate that architecture to make the plant more resilient to drought and parasitic plants are underway. These attempts often try to modulate the biosynthesis of hormones that determine root architecture and growth. Experiments are laborious and time-consuming, creating the need for simulation platforms that can integrate metabolic models and 3D root growth models and predict the effects of synthetic biology interventions on both, hormone levels and root system architectures. Here, we present an example of such a platform that is built using Mathematica. First, we develop a root model, and use it to simulate the growth of many unique 3D maize root system architectures (RSAs). Then, we couple this model to a metabolic model that simulates the biosynthesis of strigolactones, hormones that modulate root growth and development. The coupling allows us to simulate the effect of changing strigolactone levels on the architecture of the roots. We then integrate the two models in a simulation platform, where we also add the functionality to analyze the effect of strigolactone levels on root phenotype. Finally, using *in silico* experiments, we show that our models can reproduce both the phenotype of wild type maize, and the effect that varying strigolactone levels have on changing the architecture of maize roots.

## 1 Introduction

Maize is the largest crop in the world with respect to both production and consumption (OECD and Food and Agriculture Organization of the United Nations, 2021). It is a food staple to most African and Latin American countries (Nuss and Tanumihardjo, 2010; Guzzon et al., 2021). In other parts of the world, maize is used as feed for farm animals that supply meat, eggs, and dairy products, and as a source of biofuel (OECD and Food and Agriculture Organization of the United Nations, 2021). Since 2019, the average yearly global production for the crop lags 8.7 million tonnes behind its total demand (Erenstein et al., 2022). If this persists, a world food crisis is inevitable (OECD and Food and Agriculture Organization of the United Nations, 2021).

Improving maize’s root structure can contribute to crop growth, which is one of the ways to boost maize production (Paez-Garcia et al., 2015). Longer and denser roots enable the plant to forage for nutrients and water (Jaramillo et al., 2013; Tajima, 2021), making the plant less reliant on fertilizers and more resistant to drought. In principle, this could be partially achieved by manipulating the production of maize strigolactones (SLs; Rich and Ejeta, 2008; Kapulnik et al., 2011; Koltai, 2011; Ruyter-Spira et al., 2011; Arite et al., 2012; Rasmussen et al., 2012; Rasmussen et al., 2013; Sun et al., 2014; Sun et al., 2015; Gobena et al., 2017; Sun et al., 2022; Li et al., 2023; Luqman et al., 2023). SLs are plant hormones that mediate maize root development and germination of parasitic plants (Cook et al., 1966; Koltai, 2011; Sun et al., 2015). Increasing SLs levels leads to longer crown, brace, primary and seminal roots, with diminished secondary root formation through branching (Ruyter-Spira et al., 2011; Arite et al., 2012).

The greater potential for boosting maize production lies in developing countries, where drought (Fisher et al., 2015; McMillen et al., 2022) and pests (Yacoubou et al., 2021), such as *Striga hermonthica*, drag down the production(Yacoubou et al., 2021). Genetic modification of maize is the most likely means of achieving that production boost on a short timescale without the excessive use of fertilizers and insecticides that damage the environment (Liu and Stewart, 2015; Wurtzel et al., 2019; Kowalczyk et al., 2022). Still, due to the nonlinear regulatory interactions occurring at the level of gene expression and protein activity, genetic manipulation can have many unforeseen side effects (Husaini, 2022). As such, achieving a certain phenotypical goal *via* direct genetic modification is still a trial and (frequent) error process (Clark et al., 2020; Gudmundsson and Nogales, 2021), often taking too long to yield appropriate results. We need tools that accelerate the process, improve efficiency, and help minimize the probability of undesired side effects resulting from genetic manipulation. These tools should help predict the effect of alternative manipulations, allowing us to prioritize implementing the ones that are more likely to meet the desired goals.

Mathematical models are good candidates to play this role (Chandran et al., 2008; Cloutier et al., 2009; Lee and Voit, 2010; Srinivasan et al., 2019; Correa et al., 2020). By incorporating information about the genetic makeup and molecular interactions of an organism, mathematical models can simulate and predict the outcome of genetic alterations in a controlled and reproducible manner. This can help to identify potential off-target effects and unintended consequences before they occur, providing valuable information to guide decisions about genetic manipulation. Additionally, mathematical models can be used to test and refine hypotheses about gene function, providing valuable insights into the underlying biological mechanisms through *in silico* experimentation (Nijhout et al., 2015; Lucido et al., 2022). Ultimately, the use of mathematical models to predict the effects of genetic manipulation can contribute to improving the precision and efficiency of genetic engineering and help to ensure a responsible development of safe new genetic technologies.

In previous work (Lucido et al., 2022), we used mathematical models of metabolic pathways to understand how SLs biosynthesis might work in maize, and predict how that biosynthesis could be manipulated. We now intend to model and predict the effects of changing SLs levels on maize’s RSA.

There are several methodologies and platforms to model RSA. The simplest ones are based on L-Systems, where a small set of rules is used to recursively generate fractal structures that resemble roots (Prusinkiewicz et al., 1996; Leitner et al., 2010; Boudon et al., 2012). For example, RootBox is a 3D, L-system based, RSA model built in Matlab. On the other end of the complexity spectrum, some platforms utilize on ordinary and partial differential equations to generate the RSA in either mechanistic (Pagès et al., 2004; Javaux et al., 2008; Schnepf et al., 2018) or empirical way (Lynch et al., 1997; Postma et al., 2017). OpenSimRoot is an example of such a platform, allowing users to build a comprehensive and open-source functional structural plant model that allows simulating the development of a RSA by modeling root growth, and including water and nutrient uptake, carbon allocation, root plasticity, and shoot growth (Postma et al., 2017). Earlier, (Dunbabin et al., 2013), (Lobet et al., 2013) and (Schnepf et al., 2018) compared the functionality of these and other root platforms. We have now updated that comparison and further listed some of the more recent RSA modeling platforms in **Table 1** (Dunbabin et al., 2013; Lobet et al., 2013; Schnepf et al., 2018).

**Table 1:** Summary of RSA modeling platforms.

Model name	Model features	Programming Language	Numerical methods	Authors
–	RSA	Fortran	Explicit*	(Lungley, 1973)
ROOTMAP	RSA, environmental conditions, resource acquisition and allocation, water flux, solute-of-water?, containers	Turbo pascal	Explicit*	(Diggle, 1988)
Simroot	RSA, environment conditions, resource acquisition and allocation, water flux, solute flux, shoot dry mass	C++	Explicit*	(Lynch et al., 1997)
RootTyp	RSA	C, C++	Explicit*	(Pagès et al., 2004)
GRAAL-CN	RSA, environmental conditions, resource acquisitionl		Explicit*	(Drouet and Pagès, 2007)
SPACYS	RSA, environmental conditions, water flux, solute flux	C++	Explicit*	(Wu et al., 2007)
R-SWMS	RSA, environmental conditions, water flux, solute flux	C++, Fortran	Explicit*	(Javaux et al., 2008)
RootBox	RSA, environmental conditions, resource acquisition and allocation,	Matlab	L-system	(Leitner et al., 2010)
ArchiSimple	RSA, environmental conditions, shoot dry mass	C, C++	Explicit*	(Pagès et al., 2014)
OpenSimRoot	RSA, environmental conditions, resource acquisition and allocation, water flux, solute flux, shoot dry mass	C++	Explicit*, Runge-Kutta 4	(Postma et al., 2017)
CRootBox	RSA, environmental conditions, resource acquisition and allocation, water flux, containers	C++, Python	Explicit*	(Schnepf et al., 2018)
DigR	RSA, environmental conditions	C++, Java	Explicit*	(Barczi et al., 2018)

*Explicit methods calculate the values at a later time using the known values from the current time.

Environment, genetic variability, and the interaction between both affect plant development. While some of the RSA modeling platforms (Leitner et al., 2010; Postma et al., 2017; Schnepf et al., 2018) can model the interactions between root and environment, we found none that could easily be integrated with metabolic models of plant metabolism. This prevents the use of modeling to predict the macroscopic effects that changing hormone levels might have on RSAs at the macroscopic level. In previous work (Lucido et al., 2022), we modeled the biosynthesis of SLs in maize. We are now interested in coupling the SLs biosynthesis model to an RSA model and testing *in silico* the effects of changing SLs on the root.

To do so, and using Mathematica (Wolfram Research, Inc, 2022) we developed an integrated modeling platform that can simultaneously model the biosynthesis of SLs at the molecular level and the effects of changing that biosynthesis on the 3D RSA of maize. The SLs biosynthesis model predicts the amount of SLs synthesized by the plant, and this amount is one of the inputs for the RSA model. RSA model outputs are root length and lateral root branching density, among others. Here we describe the methodology used for the integrated modeling and show that we are able to mimic experimental determinations of how SLs affects RSAs and extrapolate this effect to previously undetermined SLs concentrations.

## 2 Materials and methods

### 2.1 General modeling approach and software

**Figure 1:**
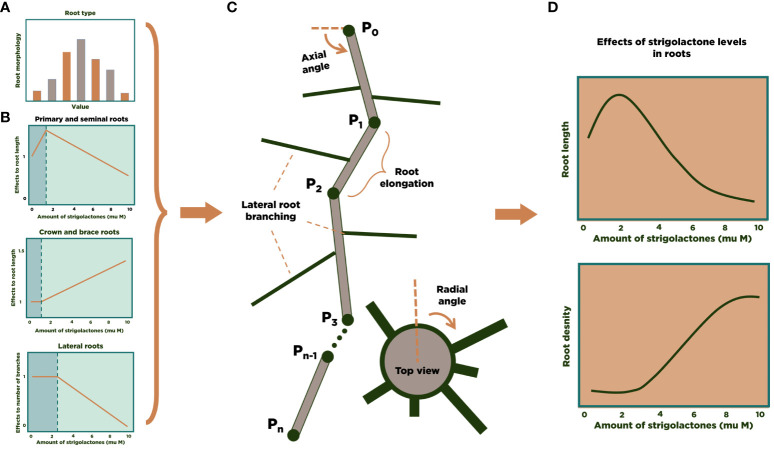
3D modeling of an RSA in maize. We calculate experimental distributions **(A)** and the effect of SL on RSA parameters **(B)** from the literature. Then, individual root strands are grown and assembled into an RSA **(C)**. After generating a large number of RSA simulations, statistical analysis of the results can be performed **(D)**.

We used Mathematica (Wolfram Research, Inc, 2022) as the simulation environment for modeling and analysis. The models developed in (Lucido et al., 2022) were implemented in a Mathematica notebook and coupled to the RSA 3D model for maize. **Figure 1** summarizes the general approach for the 3D modeling of the RSA of one maize plant. Using experimental data (Zhu et al., 2006; Hochholdinger, 2009; Jia et al., 2018; Liu et al., 2019; Liu et al., 2021) we build a statistical distribution for the characteristics of each main type of maize roots (primary, seminal, crown, brace, and secondary lateral roots for each of the other root types, **Figure 1A**). We also collected information from the literature about the influence of SLs on maize’s RSA (**Figure 1B**). The number of each type of roots in an RSA is randomly determined at the beginning of the simulation, based on the experimental distributions. Then, each main root grows (**Figure 1C**), and the simulation stochastically determines whether lateral roots branch out and for how long they grow. Finally, accumulation of enough individual RSA simulation allows us to perform statistical analysis of or modeling results (**Figure 1D**).

### 2.2 Calculating model parameters and statistical distributions for 3D maize RSA

**Table 2** summarizes the parameter values used in our 3D maize RSA model. We estimated them from the experimental data published in (Pagès et al., 1989; Hochholdinger, 2009; Arite et al., 2012). We note that n and $t_{f}$ are user-defined parameters of the simulation. The larger *n* is, the more detailed the RSA will be, and the longer the simulation will take. We set n = 200 for the simulations reported in this work. The range of values for $t_{f}$ shown in **Table 2** are simply the estimate of how long one RSA will take to be fully developed, based on (Pagès et al., 1989). **Table 3** summarizes the experimental distributions of phenotypical characteristics for each root type, as based on literature measurements (Zhu et al., 2006; Jia et al., 2018; Liu et al., 2019). We collected the data reported in the references shown in **Tables 2** and **3** and adjusted that data to the best-fit statistical distribution that describes it, using Mathematica’s FindDistribution function.

**Table 2:** Experimental parameters used to create 3D models of RSA in maize.

Notation	Description	Value	Unit	References
n	Maximum number of iterations	200*	–	
$t_{f}$	Final time	30 – 40*	days	(Pagès et al., 1989)
$k_{prim}\;$	Maximal primary root length	22.67	cm	(Arite et al., 2012)
$\;k_{sem}$	Maximal seminal root length	22.67	cm	(Arite et al., 2012)
$k_{cro}$	Maximal crown root length	22.67	cm	(Arite et al., 2012)
$k_{bra}$	Maximal brace root length	22.67	cm	(Arite et al., 2012)
$k_{lat}$	Maximal lateral root length		cm	
r	Initial growth rate	2	cm/day	(Pagès et al., 1989)
$r_{lat}$	Initial growth rate for lateral	$6.4e^{-0.8t}$	cm/day	(Pagès et al., 1989)

*Input parameter values of the model for the simulations reported in this paper.

**Table 3:** Experimental data of length and branching of maize root axes and lateral root.

Root parts	Notation	Values (Mean ± *SD*)	Unit	Distribution	References
Root axes length					
• Primary	primLen			Gamma Shape: 5 Scale: 0.22	(Zhu et al., 2006)
• Seminal	semLen			Gamma Shape: 5 Scale: 0.22	(Zhu et al., 2006)
• Crown	croLen	0.98±0.11		Normal	(Liu et al., 2019)
• Brace	braLen	1±0.38		Normal	(Liu et al., 2019)
Lateral root length					
• Primary	primLatLen	0.97±0.34		Normal	(Jia et al., 2018)
• Seminal	semLatLen	1±0.30		Normal	(Jia et al., 2018)
• Crown	croLatLen	0.95±0.17		Normal	(Jia et al., 2018)
• Brace	braLatLen	0.95±0.17		Normal	(Jia et al., 2018)
Root axes branching					
• Seminal	semBranch			Binomial Trials: 19 Probability: 0.16	(Zhu et al., 2006)
• Crown	croBranch			Gamma Shape: 91.89 Scale: 0.41	(Liu et al., 2019)
• Brace	braBranch	18.83±9.12		Normal	(Liu et al., 2019)
Lateral root branching					
• Primary	primLatBranch	6.65±0.63	$cm^{-1}$	Normal	(Jia et al., 2018)
• Seminal	semLatBranch	5.05±0.66	$cm^{-1}$	Normal	(Jia et al., 2018)
• Crown	croLatBranch	7.05±1.99	$cm^{-1}$	Normal	(Jia et al., 2018)
• Brace	braLatBranch	7.05±1.99	$cm^{-1}$	Normal	(Jia et al., 2018)

### 2.3 Building maize RSAs that simulate biological variability

Each plant has its own RSA, which depends on the interactions between genetic background, environmental cues, and other random factors (Sultan, 2000). To generate this variability, we use the distributions described in **Table 3** and assign random values drawn from those distributions each time we simulate a root. This ensures that our root models have statistical properties that match those of real plants.

We build a single maize RSA by randomly drawing its properties from the distributions in **Table 3** and deciding how coarse the time course for building that root should be. We have four modules corresponding to each root type (primary, crown, seminal, and brace roots). First, we start by defining a primary root.

Second, we find the number of crown, seminal, and brace roots from the relevant distributions in **Table 3**. The crown, seminal, and brace root modules follow the same algorithm. The primary root module follows a similar algorithm without branching. The first step of the algorithm determines the origin of all roots. Level ground is assumed to be at coordinates (0, 0, 0). Primary root and seminal roots start growing at or below the 0 coordinate in the z-direction. While shoot-borne/nodal roots are classified as either brace root if it forms above the ground or crown root if below the ground. In addition, maize has mainly 2 above ground whorls of brace root and up to 6 below ground whorls of crown roots (Hochholdinger, 2009; Liu et al., 2021). We use the distribution of the whorl distance from the study of Liu et al. (2021). We pick a random value which sets the distance between whorls of one maize root and represented this whorl distance as $w_{d}$ . Thus, the initial point of a brace root will be either (0, 0, $w_{d}$ ) or (0, 0, 2 $w_{d}$ ). For the z-coordinate of the initial point of a crown root, it will be randomly chosen from 0, - $w_{d}$ , -2 $w_{d}$ , -3 $w_{d}$ , -4 $w_{d}$ , or -5 $w_{d}$ .

Third, we randomly generate the length of each root strand, drawing this number from the relevant distribution in **Table 3**. The model picks random axial angles that will set the direction of the point subsequent to the origin of the root strand. We determine the distance between two successive points in a root strand by using an elongation function (see section 2.4) that depends on time after germination, length variability, and effect of SLs.

**Figure 2:**
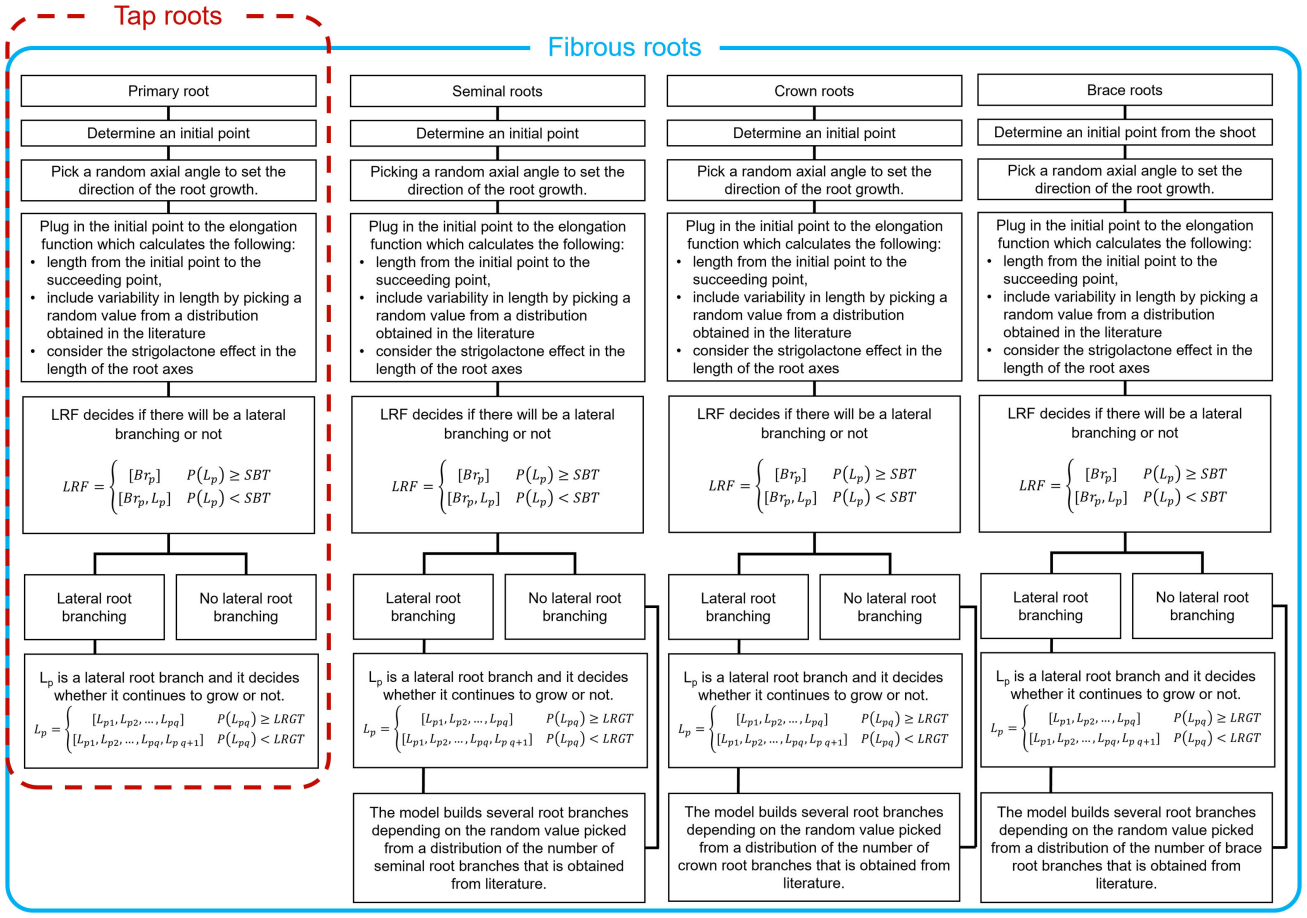
Workflow for building a 3D RSA for two different types of roots. Tap roots have only one primary root while fibrous roots have several root axes. Here we focused on modeling the root axes of maize. The procedure could be easily adapted for plant with different types of roots. Each panel have the same flow except in the last step since primary root is only one branch. The workflow starts with initial point then picks a random axial angle to decide the direction of the elongation. Next, lateral root function (LRF) simulates lateral root branching, depending on the critical parameter stop branching threshold (SBT). If there is lateral branching, then it continues to decide about elongation at every iteration, depending on lateral root growth threshold (LRGT). All other root axes will repeat the process except for primary root.

Fourth, we generate lateral roots for each main root strand. We do so by bootstrapping one thousand samples for the number of branches that sprout out of each strand and use the median value for that sample as the maximum possible number of lateral roots in the strand. Then, we use a piecewise stochastic function that decides whether there will be lateral root branching or not for each potential branching point. If there is a lateral root branch, another stochastic function determines when its growth will stop. **Figure 2** summarizes the entire process.

### 2.4 Mathematical implementation of root elongation in the 3D RSA model

**Figure 3:**
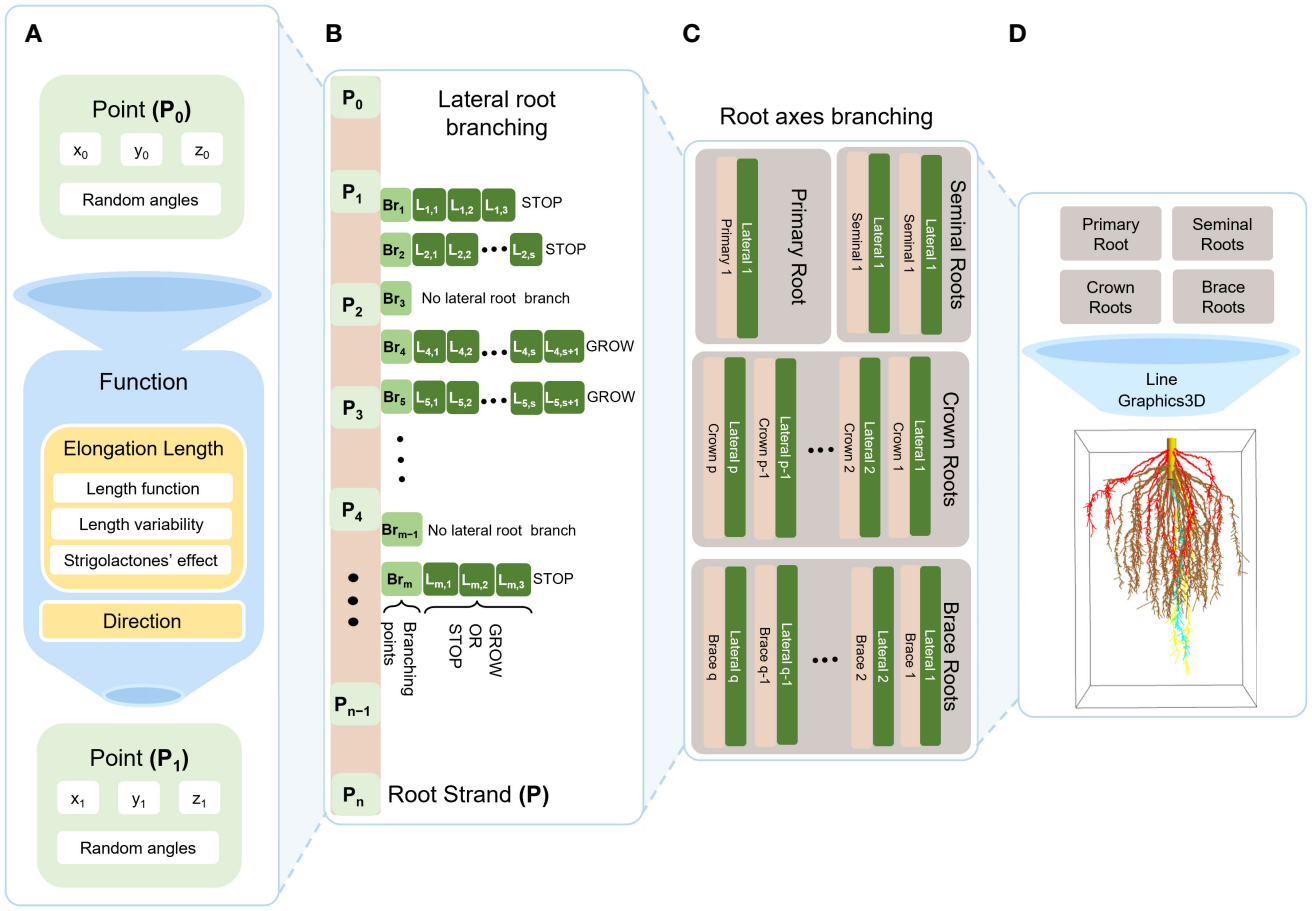
Schematic diagram of maize RSA model. First, we generate succeeding point from preceding point (initial point; *P*_0_) using a function that tells the direction and the elongation between two consecutive points which also composed of the following: length function (Pagès et al., 1989), length variability, strigolactone effects (A). Second, we continue generating points from initial point (*P*_0_) to end point (*P*_n_) where *P*_0_,…,*P*_n_ forms a single root strand P. From the root strand P, we select branching points *Br*_1_,…,*Br*_m_ in which for each point it decides whether to branch or not. If it branches, it elongates similar with the function in **(A)** with respect to lateral root growth, but here it decides whether it will continue to grow or not **(B)**. After generating a single strand with lateral roots, we repeat the same process for each respective root axis that differs on number of branches, where it is generated as random number from a distribution that we obtained from literature **(C)**. Then we use Mathematica function Line and Graphics3D to generate one root **(D)**.

**Figure 3** illustrates the growth of a maize RSA from an initial point to a 3D structure. First, we define a function that uses a point and angles to locate the subsequent point towards which the root will grow. We repeat this step until the strand is fully-grown.

We define a rule describing root elongation and branching, as shown in **Figure 3A**. A single root $P=\left\{P_{0},\;P_{1},P_{2},\ldots ,\;P_{n}\right\}$ grows from the initial point $P_{0}=\left(x_{0},y_{0},z_{0}\right)$ to the endpoint $P_{n}=\left(x_{n},y_{n},z_{n}\right)$ in a 3-dimensional space. In general, we use Equation (1) to calculate the coordinates $\left(x_{i},y_{i},z_{i}\right)\;$ of point $P_{i>0}$ :


(1) $$\begin{array}{c} x_{i}=x_{i-1}+\left(\frac{i}{n}EF\right)\left(\cos A\cos B\right)\\ y_{i}=y_{i-1}+\left(\frac{i}{n}EF\right)\left(\cos B\sin A\right)\\ z_{i}=z_{i-1}+\left(\frac{i}{n}EF\right)\left(-\sin B\right) \end{array}$$

The expression (cosAcosB) in $x_{i}$ , (cosBsinA) in $y_{i}$ , and (−sinB) in $z_{i}$ determines the direction of elongation for the root strand by using axial angles A and B . The axial angle A is set randomly between 180° and 360° while the axial angle B is set randomly between 0° to 180°.

EF is an elongation function, defined in Equation (2). It is used to calculate the length of a root strand at time $t_{i}$ , such that $t_{i}=\frac{i}{n}t_{f}$ , where i is the i^th^ iteration, n is the maximum number of iterations, and $t_{f}$ is the final time.


(2) $$EF=G\cdot LD\cdot SL_{effect}$$

We calculate *EF* by multiplying the growth function (G) in Equation (3) by a number *LD* drawn randomly from a root specific normalized length distribution (**Table 3**), and then multiplying both by a function $SL_{effect}$ (Equation 4) at the beginning of the root strand simulation. The growth function G , which was taken from Leitner et al. (2010), is defined as (3):


(3) $$G=k\;\left(1-e^{-\frac{r}{k}\;t_{i}}\right)$$

Here, k is the maximal root length, r is the initial growth rate, and $t_{i}$ stands for the iteration time step. *LD* is dimensionless and decides by what percentage the length of a specific root strand will differ from the mean length of strands from that root type. We note that the function $SL_{effect}$ , which we define below in section 2.5 describes the effect of SLs on root elongation.

### 2.5 Modeling the effect of changing external strigolactone concentration on root growth

We focus on SLs to model the effect of changing external hormone concentration on RSA. The reason for this choice was the abundance of experimental data against which to validate the model. The $SL_{effect}$ function simulates the effect of strigolactones on RSAs. Equation (4) represents $SL_{effect}$ :


(4) $$SL_{effect}=RT_{initial}+\frac{\text{Δ}\;RT}{\text{Δ}\;SL}\cdot \left(SL-SL_{initial}\right)$$

Here, $RT_{initial}$ is the wild type value for the specific root trait. For example, the root trait in our case is either root length or average number of branches. $\frac{\text{Δ}\;RT}{\text{Δ}\;SL}$ is the experimentally determined effect of changing the amount of SLs on property $RT_{\;}$ , determined by the slope in the experimental data. $SL_{initial}$ is the initial quantity of SLs, while SL is an input of the model.

**Table 4** summarizes the known effect of changing strigolactone levels on RSA. This data regard the effect of synthetic strigolactone analog GR24 on the RSA of *Arabidopsis thaliana* (Ruyter-Spira et al., 2011) and *Oryza sativa* (Arite et al., 2012), as we were unable to find quantitative information about its effect on maize RSAs. In order to extrapolate the data to maize, we normalize the effect of GR24 with respect to wild type roots of *Arabidopsis thaliana* and *Oryza sativa* and assume a similar relative effect on maize roots.

**Table 4:** Effects of strigolactones on RSA.

Strigolactone ( μM )	Effect on root length		Relative effect on root branching with respect to no added GR24
	Primary/Seminal*	Crown/Brace**	per cm of root axes*
0	1	1	1
0.01	–	1.01	–
0.1	–	0.99	–
1	–	1.10	–
1.25	1.25	–	1.08
2.5	1.2	–	1.08
5	1	–	0.73
10	0.63	1.19	0.83

*(Ruyter-Spira et al., 2011) **(Arite et al., 2012). We normalize the effects with respect to wild type roots in the absence of GR24.

Equations (1–4) are used from $t_{1},t_{2},\;\ldots ,\;t_{n}$ to generate a single root strand P .

### 2.6 Mathematical implementation of lateral root branching in the 3D RSA model

Lateral roots branch out from primary, seminal, crown, and brace roots. Lateral root branching is often quantified by counting the number of lateral root branches per cm of the axis root. K.-P. Jia et al. (2018) reported that there is variation in the number of lateral roots per cm for each type of main roots. We consider this in building a RSA, as illustrated in **Figure 3B**.

The simulation of branching in a root strand starts by drawing a random number B from the distributions in **Table 3**. We multiply this number by the length of the strand and obtain the maximum number of possible branches, $\left\{Br_{1},\;Br_{2},\;\ldots ,\;Br_{m}\right\}$ , in that strand. $Br_{p}$ is a point in 3D space:


(5) $$Br_{p}=\begin{array}{cc} \left(x_{p},\;y_{p},z_{p}\right), & p=1,\;2,\ldots ,\;m. \end{array}$$

From each branching point, the lateral root function (LRF) stochastically decides if a lateral branch will be formed or not by drawing a random number between 0 and 1and comparing that number to the probability of branching defined in Equation (6).


(6) $$LRF=\{\begin{array}{cc} \left[Br_{p}\right], & P\left(L_{p}\right)-\;SBT\geq 0\\ \left[Br_{p},\;L_{p}\right] & P\left(L_{p}\right)-\;SBT<0 \end{array}$$

In Equation (6), each branching point $Br_{p}$ will generate $P\left(L_{p}\right)$ , a random value between 0 and the maximum number of lateral root branches, denoted as MaxL . Lateral root branching depends on whether $P\left(L_{p}\right)$ is greater than the stop branching threshold ( SBT ) threshold, such that $SBT=MedL\cdot SL_{effect}$ . MedL is the median of 1000 generated random value from the distribution in **Table 3** and $SL_{effect}$ was defined earlier, in Equation (4).

If, according to Equation (6), a lateral root branch forms, then $Br_{p}$ will be connected to the set of points $L_{p}$ , as illustrated in **Figure 3B**. The length of the lateral root $L_{p}$ is determined based the piecewise function defined in Equation (7). We use this equation to calculate the probability of growth for the lateral root $P\left(L_{pq}\right)$ and the lateral root growth threshold ( LRGT ).


(7) $$L_{p}=\{\begin{array}{cc} \left[L_{p1},\;L_{p2},\ldots ,L_{p\;q}\right], & P\left(L_{p\;q}\right)-\;LRGT\geq 0\\ \left[L_{p1},\;L_{p2},\ldots ,L_{p\;q},L_{p\;q+1}\right] & P\left(L_{p\;q}\right)-\;LRGT<0 \end{array}$$

$P\left(L_{pq}\right)$ is a random value from 0 to 1 and LRGT is a random value from 0.4 to 0.9 which we choose to simulate biological variability in lateral roots, as there is no strong evidence about the effect of strigolactones to lateral root elongation (Ruyter-Spira et al., 2011; Arite et al., 2012). The condition in Equation (7) states that when $P\left(L_{pq}\right)$ is above the threshold then the lateral root stops growing at $L_{pq}$ , otherwise it will continue to grow up to $L_{p\;q+1}$ .

As in Equation (1), we define the points of the lateral root $L_{p}$ as


(8) $$\begin{array}{c} L_{pq}=\begin{array}{cc} \left(u_{pq},\;v_{pq},w_{pq}\right), & q=1,\;2,\ldots ,\;s \end{array}\\ u_{pq}=x_{p}+\left(G_{lat}\cdot LLD\right)\cos\left(A\pm R\right)\cos\left(B\pm R\right)\\ v_{pq}=y_{p}+\left(G_{lat}\cdot LLD\right)\cos\left(B\pm R\right)\sin\left(A\pm R\right)\\ w_{pq}=z_{p}+\left(G_{lat}\cdot LLD\right)(-\sin\left(B\pm R\right)). \end{array}$$

In Equation (8), $x_{p},\;y_{p},\;z_{p}$ are the coordinates of branching point $Br_{p}$ from (5), which is the initial point of the lateral root branch $L_{p}$ . The expressions cos(A±R)cos(B±R) in $u_{pq}$ , cos(B±R)sin(A±R) in $v_{pq}$ , and −sin(B±R) in $w_{pq}$ determine the direction of the lateral root $L_{p}$ in three-dimensional space, in an analogous process to that described for Equation (1), adding/subtracting R to the axial angles. We remind the reader that angles A,B are the same axial angles from the root axis P where the branching point $Br_{p}$ belongs. In this context, R represents the radial angle of $L_{p}$ , set to be a random angle between 0° and 180°. Lastly, $G_{lat}\cdot LLD$ determines the length of $L_{p}$ per iteration where $G_{lat}$ is the same function in Equation (3) but with a different initial growth rate $r_{lat}$ and a maximal root length $k_{lat}$ than those specified for the lateral root. *LLD* is a random number drawn from the appropriate normalized lateral root length distribution defined in **Table 3** and works in a similar way as *LD* in Equation (2).


$$G_{lat}=k_{lat}\left(1-e^{-\frac{r_{lat}}{k_{lat}}t}\right)$$

(9) $$r_{lat}=6.4e^{-0.8t}$$

Equations (5–9) are iteratively used to simulate all branching points in Br . The final list is merged to generate a single root strand with lateral branches.

## 3 Results

### 3.1 Modeling maize RSAs

As described in the methods section, we used Mathematica to simulate the growth of a Root System Architecture (RSA) for a maize plant (**Supplementary Data Sheet S1**). The use of random number generation ensures that, while all RSA have similar phenotypical characteristics, each RSA is unique. We illustrate this in **Supplementary Data Sheet S1**, where we present 5 examples of RSA that were generated using the same initial parameters.

**Figure 4:**
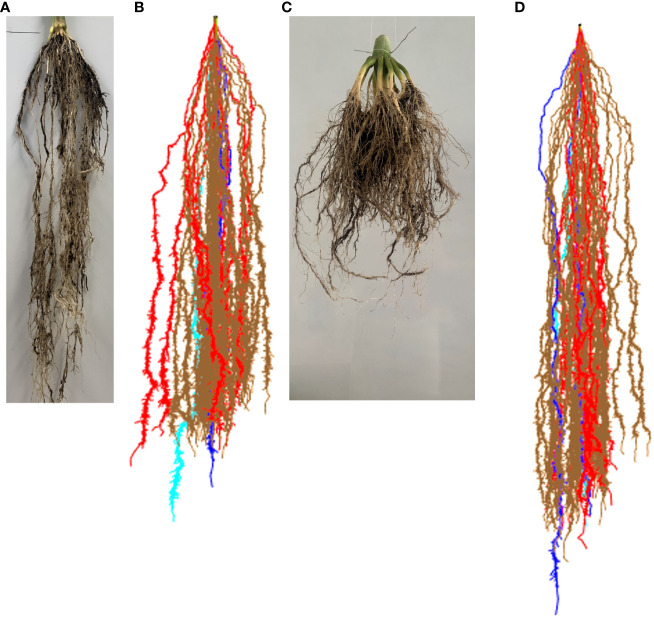
Comparison of real maize root and our model results. **(A)** Maize roots at 75 days after seeding. **(B)** Simulated maize RSA at 75 days after simulation start. **(C)** Maize roots at 120 days after seeding. **(D)** Simulated maize RSA at 120 days after simulation start.

We further validated the ability of our modeling methodology to reproduce RSAs and their development. We took photos of two individual maize roots at two different developmental stages. The plants are South African elite white maize variety M37W and were grown in University of Lleida. Then, we simulate a RSA with our modeling methodology and take a snapshot of the model at the same two developmental stages. **Figures 4A**, **B** show an RSA at 75 days after seeding, while **Figures 4C**, **D** show RSAs at 120 days after seeding. In both cases, our model-generated RSA is similar to the real RSA. We also provide a short animation of a growing root in **Supplementary Video S1**.

**Figure 5:**
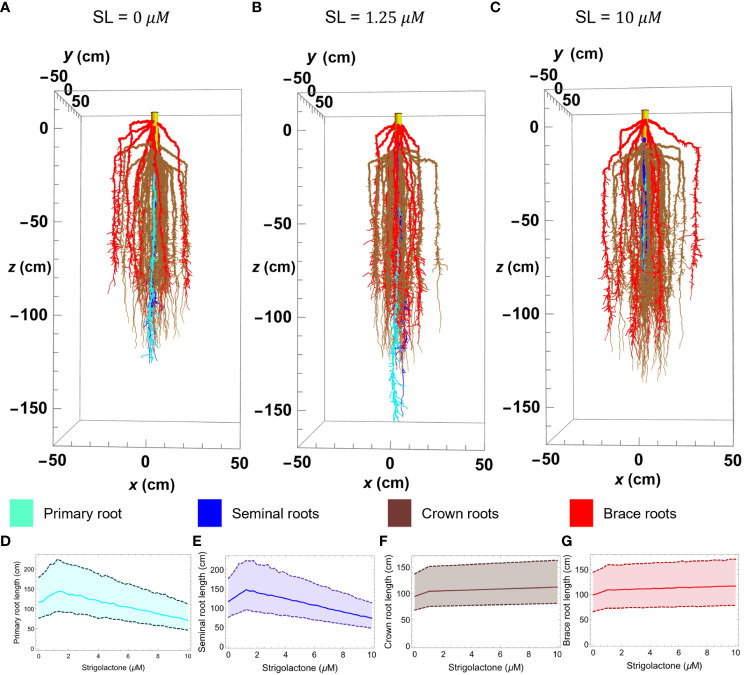
Maize root 3D simulation results of the RSA model in Mathematica with varying strigolactone quantity; 0 *μM* **(A)**, 1.25 *μM* **(B)**, 10 *μM* **(C)**. Plots **(D–G)** show the changes in length of primary, seminal, crown, and brace roots, respectively, as strigolactone quantity changes from 0 to 10 μM. The plot is obtained using the median of 100 simulated roots with 99% confidence interval. The color corresponds to different root types; primary (cyan), seminal (yellow), crown (brown), and brace (red).

**Figure 6:**
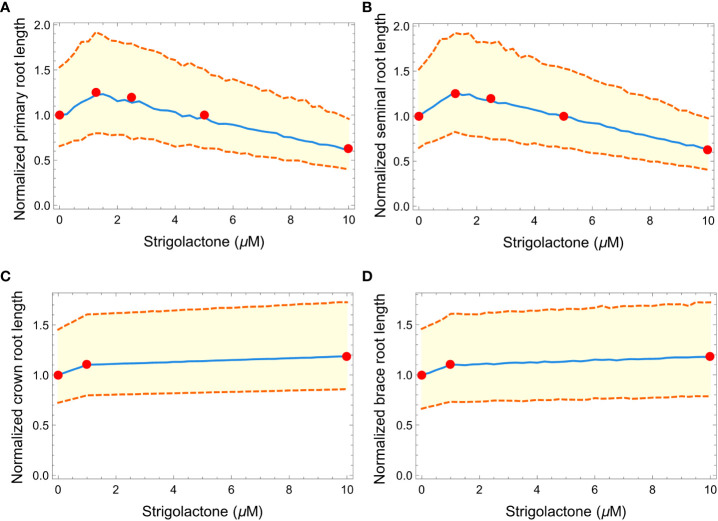
Plots showing the effects of increasing strigolactone quantity (from 0 to 10 *μM*) to the normalized length of primary **(A)**, seminal **(B)**, crown **(C)**, and brace **(D)** roots in maize. Red dots represent the experimental results from Ruyter-Spira et al. (2011) and Arite et al. (2012). The blue line is the median of 100 roots while the orange dashed lines represent the 99% confidence interval.

To ensure that, in addition to being able to reproduce RSAs appearance, the modeling methodology can indeed reproduce the statistical properties of real RSAs, we generated a set of 100 wild type maize RSAs. We then calculated the median length and the number of lateral roots per cm of each root axis for each of the 100 RSAs. Subsequently, we compared our results to experimental data (Ruyter-Spira et al., 2011; Arite et al., 2012). We present the results of the comparison in **Figures 5** and **6**. In all cases, the average primary, seminal, crown, and brace root lengths are within 1% of the experimental values.

### 3.2 Modeling the effect of SLs on maize RSAs

In previous work (Lucido et al., 2022) we modeled the biosynthesis of SLs in maize. The supplementary materials of that reference contain the metabolic models that permit simulating the biosynthesis of both strigol-type and orobanchol-type of strigolactones. The output of those models can then be used as input for the RSA modeling platform (**Supplementary Data Sheet S2**). To model the effect of changing SLs concentrations on maize RSAs we repeat the simulations described in section 3.1, generating sets of RSAs containing 100 root systems each, at concentrations of SLs that range from 0.25 to 10 μM with increment of 0.25. In total, we generate 40 sets of 100 maize RSAs. We then calculate the median of the root length of each root axis and the number of lateral roots per cm of each root axes for each set. We normalized these results with respect to the wildtype, growing at physiological levels of SLs. Then we compare our results to experimental data (Ruyter-Spira et al., 2011; Arite et al., 2012). We present the results for the comparison in **Figures 5**, **6**.

**Figure 5** illustrates the effect of varying SLs levels on the length of different root types in our simulated RSA models. To create that figure we generated three architectures at different SLs concentrations (**Figures 5A–C**), using the parameter values in **Table 2** and obtaining 2 seminal root axes (blue), 39 crown root axes (brown), and 12 brace root axes (red) from the distribution in **Table 3**. To facilitate visualizing the main root axes, we limit lateral root growth in the simulations by increasing the lateral root growth threshold (LRGT) to 0.7. By comparing **Figures 5A** (wild type maize and physiological SLs levels) and 5B (SL= 1.25 μM) we see that increasing SLs amounts in this range leads to the elongation of seminal (brown) and primary (cyan) roots. In contrast, when the amount of SLs increases to 10 μM (**Figure 5C**) we observe a decrease in the length of those roots.

The properties of 100 RSAs models generated at each of those SLs concentrations, show that the models statistically follow a similar trend for the effect of SLs concentration on the length of primary and seminal roots, respectively.

In contrast, the influence of SLs on the length of crown and brace roots is monotonic. The length of both types of these roots increases in a way that matches those observed for seminal and primary roots when SLs concentrations are below 1.25μM. When the concentration of SLs increases from 1.25μM to 10 μM the length of crown and brace roots increases only slightly (**Figures 5F, G**).

We also tested if our simulated RSA could reproduce the effects of changing the concentration of the SLs analog GR24 on plant RSAs. **Figure 6** summarizes the results and shows how our simulated RSAs compare to real RSAs. Because the experimental data were obtained from *Arabidopsis thaliana* (Ruyter-Spira et al., 2011) and *Oryza sativa* (Arite et al., 2012), we normalized the length of each plant (*Arabidopsis thaliana*, *Oryza sativa* and simulated *Zea mays* RSAs) by the length of the RSA when 0 μM of GR24 are added to the medium, to make the effects quantitatively comparable across species. **Figure 6** clearly shows that our simulated RSAs reproduce the trend of the experimental results for all types of roots.

### 3.3 Effects of GR24 on lateral root branching

**Figure 7:**
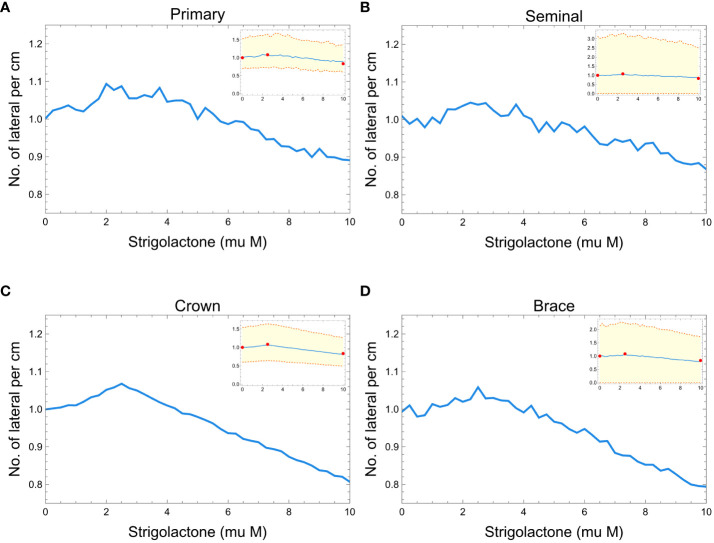
Plots showing the effects of strigolactones (within the range of 0 to 10 *μM*) to the normalized lateral root branching for primary **(A)**, seminal **(B)**, crown **(C)**, and brace **(D)** roots of maize. Red dots represent the experimental results from Ruyter-Spira et al. (2011). The blue line is the median of 100 roots while the orange dashed lines represent the 99% confidence interval.

**Figure 7** shows the comparison between our model results of lateral root branching per cm of root axis and experimental results from the study of Ruyter-Spira et al. (2011). In all the plots, we can observe minimal changes in the normalized lateral root branching per cm of the root axis within the 0 to 10 μM of SLs. **Figures 7A–D** show that we can barely notice the changes in the density of lateral root branches as the concentration of SLs changes. Still, while the effect is small, we see that the simulated RSAs replicate the trend observed in real RSAs (red dots in **Figure 7**).

## 4 Discussion

Altering a plant’s genes to enhance crop quality affects metabolism, which subsequently influences the plant’s architecture and phenotype. Thus, it is crucial to have simulation platforms that can concurrently model changes in gene expression, protein activity, metabolite concentrations, and their effects on the overall plant structure. There are several modeling platforms that can simulate RSAs, but only a few of them can simulate how environmental interactions and nutrient uptake affect RSAs (**Table 1**). None of these platforms can simulate how genome manipulation affects metabolism and how this affects RSAs. The work reported here and elsewhere (Comas et al., 2016; Pereira et al., 2018; Basallo et al., 2023) serves as a proof of principle that such multilevel modeling efforts are possible.

Mathematica, with its versatility and extensive function library, offers a suitable environment for developing a platform to perform such multilevel simulations and analysis. Given Mathematica’s ability to easily implement metabolic and gene circuit models (Comas et al., 2016; Pereira et al., 2018; Basallo et al., 2023), the current manuscript illustrates how to model the effects of different SLs levels on maize root system properties through simulated growth of 3D RSAs. Leveraging our prior work on modeling SLs biosynthesis (Lucido et al., 2022), here we extended the model to simulate the effects of varying SLs concentrations on maize RSAs.

How do these phenotypical characteristics of roots affect plant performance? This performance strongly relates to, among other things, the capacity of plants to acquire resources from the environment and use those resources to grow (Lynch, 2007). Strigolactone, despite being a germination stimulant, can be utilized as a beneficial hormone that alters the RSA towards longer root axes and denser lateral root branches. On the one hand, longer root axes allow root exploration to deeper soil, where additional water sources become accessible to the plant. On the other hand, higher root densities improve the uptake of mineral nutrients (Tajima, 2021). Hence, overall, a wider area for root exploration benefits resource acquisition and makes plants more resilient to drought and less reliant on fertilizers. This coincides with the goal of the second Green Revolution that aims to make resilient crops that can still be productive despite harsh environmental conditions (Lynch, 2007).

Our 3D RSA model enables direct simulation, analysis, and visualization of the effects of changing SLs concentrations on the root system architecture. This, in turn, allows an indirect inference of the effect of SLs levels on the ability of the RSA to acquire resources for growth. Our simulation results suggest that SLs amounts lower than 2.5μM promote elongation of several main root axes (namely primary, seminal, crown, and brace) and lead to more dense lateral root branching. This is consistent with the known experimental results discussed throughout the paper (Ruyter-Spira et al., 2011; Arite et al., 2012). Maize with strigolactone quantity that ranges from 0.25 to 2.5μM shows the longest primary and seminal root and more lateral root branches. While, increasing strigolactones up to 10μM results in shorter primary and seminal roots, lesser lateral root branching but slightly longer nodal (crown and brace) roots (see **Figures 5**-**7**). These results highlight the potential of modeling to analyze multilevel effects of genetic modifications on the metabolism, physiology, and architecture of plants.

The work presented here does have several limitations; however, each limitation presents an opportunity for methodological advancements that can facilitate the exploration of intriguing biological questions. Firstly, our maize RSA modeling platform is still in its early stages compared to platforms like OpenSimRoot (Postma et al., 2017). Still, our methodologies are distinct and our approach implements a degree of stochasticity on root growth that mimics real plants. In addition, our approach enables direct simulation of metabolite effects, such as SLs, on RSA growth, which is something you cannot yet do in other root growth simulation platforms.

Secondly, our current study focuses solely on the impact of changing SLs concentrations on RSA architecture. We chose to model the effect of SLs on RSA growth because it is well established that SLs influences various parameters in RSA and an established model for the biosynthesis of SLs already existed (Lucido et al., 2022). As such, we could illustrate how we could simultaneously model the dynamic behavior of SL biosynthesis and the effect of changing SLs amounts on RSA growth and validate our approach by comparison to experimental data. However, many other factors, such as the environment and other intrinsic plant signals, also influence this architecture (Smith and Smet, 2012; Khan et al., 2016; Lombardi et al., 2021; Maqbool et al., 2022). Known intrinsic signals encompass hormones like auxins, brassinosteroids, cytokinins, ethylene, abscisic acid, and signaling peptides (Smith and Smet, 2012; Khan et al., 2016; Lombardi et al., 2021; Maqbool et al., 2022). This limitation presents an opportunity to expand our methodology and include these additional factors and their interactions (He et al., 2023). Expanding the model to simulate the effects of other hormones requires suitable experimental data. Similarly, we can extend our methodology to account for environmental effects by simulating nutrient and water uptake, interactions with the environment and other organisms, and above-ground plant growth. In fact, SLs serve as vital exudates that can trigger hyphal branching in arbuscular mycorrhizal fungi (Steinkellner et al., 2007; De Cuyper and Goormachtig, 2017; Saleem et al., 2018; Olanrewaju et al., 2019). Including SLs in our model facilitates the integration of fungi and simulating their symbiotic relationship with plants, which enhances plant nutrient uptake, particularly phosphorus (Wang, 2023).

Thirdly, our model is currently only applicable to maize RSAs, opening the door to methodological developments that would require additional work to implement other types of plant RSAs (Maqbool et al., 2022).

Finally, the availability of data for calibrating our model’s implementation is limited. This presents an opportunity to use modeling to elucidate root development aspects and prioritize experiments in synthetic biology and plant biology. By simulating RSA development across various parameter ranges, we can formulate *in silico* RSAs that generate hypotheses about the correct parameter ranges. Physiological parameter ranges should yield simulated RSAs similar to real ones, enabling the prioritization of experiments and accelerating the development of new plant varieties.

## Data availability statement

The original contributions presented in the study are included in the article/**Supplementary Material**. Further inquiries can be directed to the corresponding author.

## Author contributions

AL: Conceptualization, Data curation, Formal analysis, Investigation, Methodology, Software, Validation, Visualization, Writing – original draft, Writing – review & editing. FA: Data curation, Investigation, Validation, Visualization, Writing – review & editing. OB: Formal analysis, Validation, Writing – review & editing. AE: Investigation, Methodology, Writing – review & editing. AM-S: Conceptualization, Formal analysis, Writing – review & editing. EV: Conceptualization, Formal analysis, Writing – review & editing. AS: Conceptualization, Formal analysis, Writing – review & editing. RA: Conceptualization, Formal analysis, Funding acquisition, Investigation, Methodology, Project administration, Resources, Supervision, Validation, Writing – original draft, Writing – review & editing.
