# Supplementary figures and images for: Modeling the effects of strigolactone levels on maize root system architecture
Source: Front Plant Sci. 2024 Jan 11;14:1329556. doi: 10.3389/fpls.2023.1329556 (PMC10808495; doi:10.3389/fpls.2023.1329556)

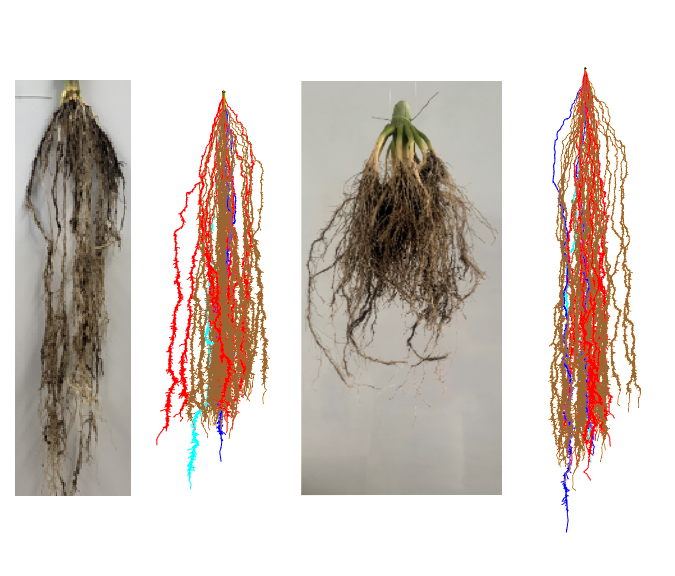

Supplement: Supplementary file 1 [file DataSheet_1.zip › Supplementary Data File S1/Figure4.tif]

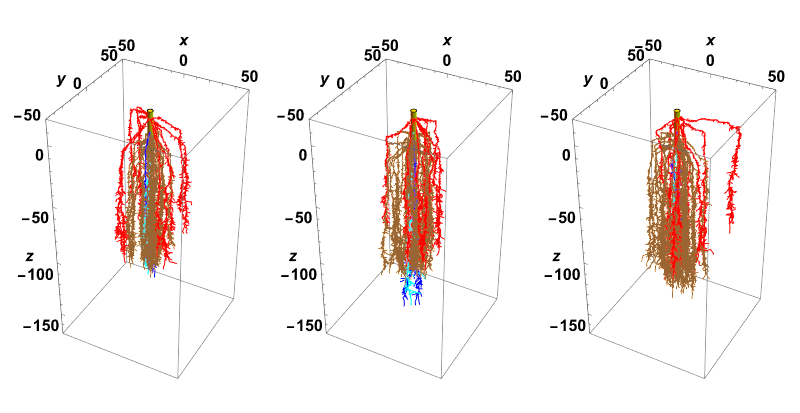

Supplement: Supplementary file 1 [file DataSheet_1.zip › Supplementary Data File S1/Figure5.tif]

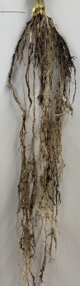

Supplement: Supplementary file 1 [file DataSheet_1.zip › Supplementary Data File S1/real_maize1.tif]

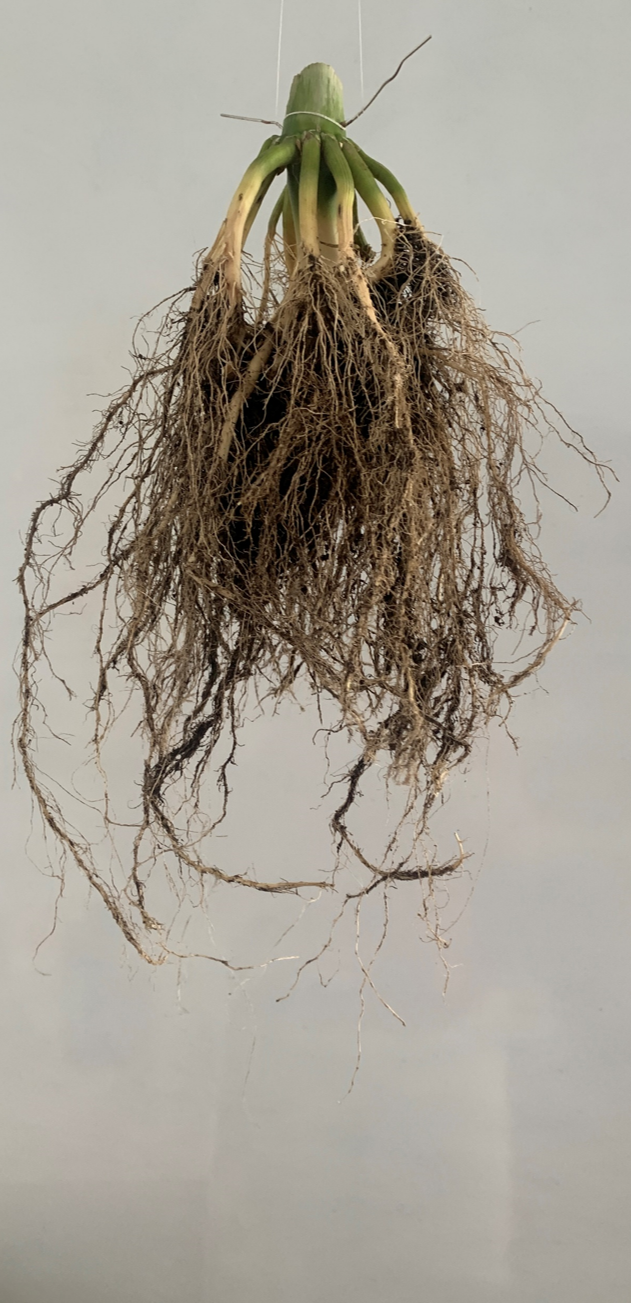

Supplement: Supplementary file 1 [file DataSheet_1.zip › Supplementary Data File S1/real_maize2.tif]

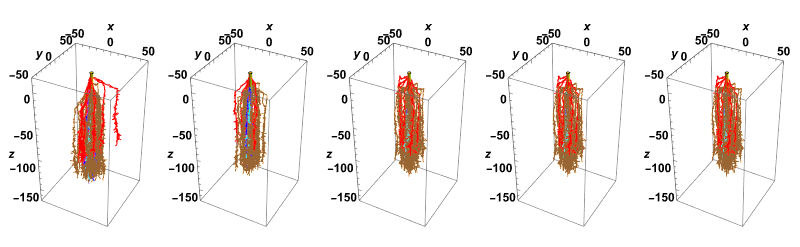

Supplement: Supplementary file 2 [file DataSheet_2.zip › Supplementary Data File S2/EffectkoRoot.tif]

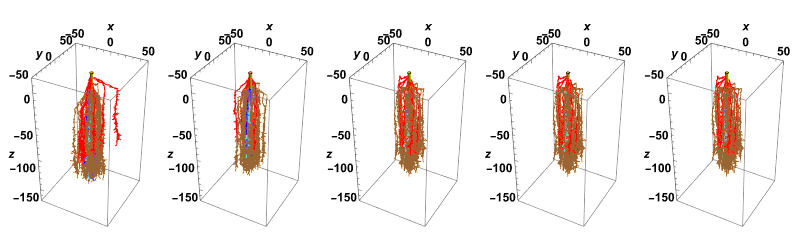

Supplement: Supplementary file 2 [file DataSheet_2.zip › Supplementary Data File S2/EffectkRoot.tif]
